# Vision-Capable LLMs in Microsurgery: A Blinded Comparison of Two AI Models with Expert Microsurgeons in the Appraisal of 200 Experimental Anastomoses

**DOI:** 10.3390/medsci14020235

**Published:** 2026-05-02

**Authors:** Victor Esanu, Horatiu Alexandru Colosi, Stefan Agoston, Elisa Marziali, Radu Alexandru Ilies, Lorena Maria Hantig, Claudia Mihaela Paun, Alexandra Ioana Stoia, Alexia Onaciu, Iulia Cezara Pop, Cristina Maria Boznea, Ana-Maria Vartolomei, Farran Moustafa, Clemens Dirven, George Calin Dindelegan, Victor Volovici

**Affiliations:** 1 Center for Complex Microvascular Surgery, Erasmus Universitair Medisch Centrum, 3015 GD Rotterdam, The Netherlands; v.esanu@erasmusmc.nl (V.E.); v.volovici@erasmusmc.nl (V.V.); 2 Department of Experimental Microsurgery, Simulation and Experiment Center, Iuliu Hațieganu University of Medicine and Pharmacy, 400347 Cluj-Napoca, Romania; agoston.stefan@elearn.umfcluj.ro (S.A.); marziali.elisa@elearn.umfcluj.ro (E.M.); ilies.radu.alexandru@elearn.umfcluj.ro (R.A.I.); hantig.lorena.maria@elearn.umfcluj.ro (L.M.H.); paun.claudiamihaela@elearn.umfcluj.ro (C.M.P.); ioana.alex.stoia@elearn.umfcluj.ro (A.I.S.); onaciu.alexia@elearn.umfcluj.ro (A.O.); pop.iulia.cezara@elearn.umfcluj.ro (I.C.P.); boznea.cristina.maria@elearn.umfcluj.ro (C.M.B.); vartolomei.ana.maria@elearn.umfcluj.ro (A.-M.V.); 3 Department of Vascular Surgery, Second Surgical Unit, Emergency County Hospital Cluj, 400006 Cluj-Napoca, Romania; farran_moustafa@elearn.umfcluj.ro; 4 Department of Medical Informatics and Biostatistics, Iuliu Hațieganu University of Medicine and Pharmacy, 400349 Cluj-Napoca, Romania; 5 Faculty of Medicine, Iuliu Hațieganu University of Medicine and Pharmacy, 400347 Cluj-Napoca, Romania; 6 Department of Neurosurgery, Erasmus Universitair Medisch Centrum, 3015 GD Rotterdam, The Netherlands; c.dirven@erasmusmc.nl; 7 Department of General Surgery, Iuliu Hațieganu University of Medicine and Pharmacy, 400347 Cluj-Napoca, Romania; 8 First Surgical Unit, Emergency County Hospital Cluj, 400006 Cluj-Napoca, Romania

**Keywords:** artificial intelligence, large language model, microsurgery, microvascular anastomosis, experimental surgery

## Abstract

**Background/Objectives**: Objective end-product assessment of microsurgical anastomoses is intensive and partly subjective. Vision-capable large language models (LLMs) may enable standardized image-based scoring, but their agreement with expert assessment remains uncertain. **Methods**: We studied 200 end-to-end femoral artery anastomoses, performed on chicken legs by novice, intermediate, and experienced microsurgeons. Images were scored independently by two blinded expert panels; disagreements were adjudicated by a third senior reviewer to establish expert consensus. Two LLMs, ChatGPT 5.2 Thinking Extended and Gemini 3.1 Pro, were evaluated using the exact same prompt and rubric. Each image was analyzed three times per model. Final scores were aggregated by median for numeric items and majority vote for categorical items. The primary endpoint was exact-match agreement with expert consensus. Agreement within ±1 was also assessed for numeric items. Agreement was measured using simple percentage agreement, Light’s kappa, and Krippendorff’s alpha; Bland–Altman analysis was used for numeric count items. **Results**: LLM 1 achieved a higher overall exact-match agreement than LLM 2 (0.659 vs. 0.539). Both models performed better on categorical than numeric items (0.713 vs. 0.610 and 0.651 vs. 0.445, respectively). LLM 1 showed the greatest advantages for gaps, knots, oblique stitches, and wide bites. Krippendorff’s alpha was positive for most endpoints with LLM 1, whereas LLM 2 showed negative values throughout. Allowing a ±1 tolerance for numeric items greatly improved agreement, suggesting only minor counting discrepancies, from 0.610 to 0.900 for LLM 1 and from 0.445 to 0.826 for LLM 2. **Conclusions**: Under a constrained scoring workflow, LLMs partially approximated intraluminal microsurgical end-product scoring. LLM 1 outperformed LLM 2, but agreement remained insufficient to replace the expert assessment entirely. These models can be assistive tools within a human-in-the-loop framework.

## 1. Introduction

Microvascular anastomosis is a technically demanding skill that is usually learned in simulation settings before it is performed clinically [1,2]. Objective end-product assessment of microsurgical technique remains challenging because of the subjectivity of evaluation and the lengthy process involved in analyzing each error in particular [3,4]. Borderline findings may be interpreted differently even by experienced microsurgeons, and agreement can be further affected by image quality or by individual interpretations for defining an error [5,6]. As a result, microsurgical evaluation often requires more than one reviewer, which increases time, cost, and difficulty of scaling assessments across large trainee cohorts or multicenter studies [7].

This dependence on expert review can become a practical bottleneck. Detailed scoring requires careful inspection of multiple intraluminal features and consistent application of the same criteria across cases [8,9,10]. In high-volume training settings, where many anastomoses may need to be reviewed, this level of assessment is difficult to sustain. Feedback may therefore be delayed or reduced to simpler endpoints, such as patency testing like the milking test, rather than a structured analysis of each error. This limitation is particularly relevant in microsurgical training, where detailed feedback on specific technical errors has been shown to accelerate skill acquisition more effectively than global assessments alone [5]. A tool capable of providing feedback could therefore complement expert review and enhance the training process, especially when access to experienced evaluators is limited.

Recent advances in artificial intelligence, particularly vision-capable large language models (LLMs), have created a practical way to test images without having to build custom computer vision pipelines [11,12]. These models are easy to access and deploy, and potentially well-suited for standardized scoring workflows [13,14]. If they can approximate expert assessment with reasonable reliability, they could reduce reviewer workload and improve consistency across training environments [15]. However, their real performance, including stability across repeated runs and agreement with expert evaluation, still needs to be tested. Moreover, most AI approaches in microsurgery have focused on procedural aspects such as instrument tracking and motion analysis, rather than on structured end-product scoring of completed anastomoses. Whether general-purpose vision-capable LLMs can assess intraluminal findings with the consistency required for training feedback remains an open question.

In this study, we compared two state-of-the-art vision-capable LLMs with blinded expert human analysis for end-product error scoring of intraluminal images from experimental microvascular anastomoses. To our knowledge, this is the first study to directly compare general-purpose vision-capable LLMs with blinded expert panels for structured intraluminal end-product scoring of microvascular anastomoses. Unlike previous approaches that relied on custom computer vision models trained on specific datasets, the present design used commercially available LLMs with a standard prompt and rubric, making the methodology immediately reproducible by other research groups.

## 2. Materials and Methods

A comparative rater agreement study was conducted on 200 end-to-end (ETE) femoral artery anastomoses performed on chicken legs by novice, intermediate, and experienced microsurgeons in two centers. Operator experience was self-declared at enrollment and confirmed with the Anastomosis Lapse Index (ALI) score derived from their anastomosis [8]. Exact stratification of participants was not an aim of the study; instead, it was used to document the range of experience levels represented in the cohort and to confirm that anastomoses of varying technical quality were included in the analyzed image set. All procedures were conducted under institutional approval and in accordance with applicable regulations (MEC-2024-0656, AVZ247, date of approval: 7 September 2022). Informed consent was obtained from all participating microsurgeons prior to enrollment and before any study-related procedures.

**Figure 1:**
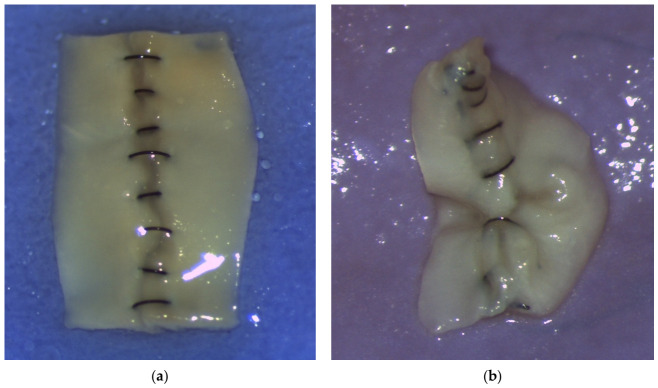
(**a**,**b**) Intraluminal standard images.

After completion, each anastomosis was opened longitudinally and photographed under the microscope from the intraluminal (intimal) view. From this perspective, the external knots were not visible, only the intraluminal stitch loops. For each anastomosis, the highest-quality intraluminal image was selected for scoring, blinded to operator identity, based on clarity of the anastomotic line, adequate focus, minimal glare, and sufficient magnification, as shown in Figure 1. A total of 203 eligible images were selected, which were rounded to 200 to facilitate reporting. No a priori sample size calculation was performed. The goal was to obtain as many anastomoses as possible to increase statistical significance.

Each image was scored independently by two blinded panels of experienced microsurgeons using a predefined rubric based on our systematic review of microsurgical errors described in the literature, which can be visualized from an intraluminal image [10]. Each panel included 2 experienced microsurgeons. Disagreements were adjudicated by a senior reviewer, resulting in a single dataset for the human analysis.

The scoring rubric included predefined categorical and numeric items, as well as a “Not assessable” option for cases in which image visibility did not allow reliable scoring. The rubric was operationalized in an Excel file and added to the LLM instructions. Model outputs were restricted to a single standardized one-row table per image with fixed columns and response options. The full scoring rubric is provided in Supplementary File S1. The rubric comprised six numeric count items (number of knots, gaps, oblique stitches, wide bites, partial-thickness bites, and tears) and six categorical items (tension, wall catch, bite size, overlap grade, thread in lumen, and line disruption). Numeric items required the rater to count the number of stitches exhibiting a given error, while categorical items required classification into predefined response options. Each definition included explicit threshold criteria to reduce ambiguity; for example, a bite was classified as wide only if the puncture-to-edge distance exceeded 1.70 times the median bite depth in that image, and a gap was defined as a segment where inter-stitch spacing was approximately 1.5–2 times the typical spacing. All anastomoses were performed under standardized operating conditions across both centers. Leica (Leica Microsystems GmbH, Wetzlar, Germany) and Zeiss (Carl Zeiss Meditec AG, Jena, Germany) surgical microscopes equipped with their integrated adjustable coaxial LED illumination were used, at a fixed magnification of 25×. Standard microsurgical instrumentation and 8-0 monofilament suture material were used throughout. No differences in instrumentation, lighting, or magnification settings were introduced across centers or operators, ensuring comparable intraluminal imaging conditions for all cases.

Taking into consideration the current benchmarks for the best and most used LLMs, two were chosen and evaluated between 24–27 February 2026:

- LLM 1: ChatGPT 5.2 Thinking Extended (OpenAI, San Francisco, CA, USA) [16].
- LLM 2: Gemini 3.1 Pro (Google LLC, Mountain View, CA, USA) [17].

Both models were run using the same rubric and general prompt created with prompt engineering techniques for LLMs [18]. To avoid contextual carryover, each image was evaluated in its own conversation (one photo per conversation). In ChatGPT, images were processed within projects organized in batches of 20 images per project. In Gemini, a dedicated Gem configuration mirrored the same prompt and rubric, and each image was evaluated in the same manner in a separate chat. The exact prompts and project organization are provided in Supplementary Table S2. The final prompt and rubric were fixed before scoring began, and no prompt changes were made after evaluation started. No external plugins or custom computer vision code were used. The human expert panels were blinded to each other’s assessments and to the LLM outputs. Similarly, each LLM evaluation was conducted without access to the human scores or to the other model’s results. This triple-blinding design ensured that all three rating sources—LLM 1, LLM 2, and the adjudicated human consensus—were fully independent, thereby minimizing the risk of confirmation bias or cross-contamination of assessments.

Because generative LLM outputs may vary across repeated runs even when the same image and prompt are used, each image was analyzed three times independently by each model in separate conversations. A single final value then resulted for each item by aggregating the three runs: the median for numeric count items and the majority vote for the categorical items. This aggregation approach was chosen to minimize the influence of outlier responses from individual runs while preserving the central tendency of each model’s output. For numeric items, the median was preferred over the mean because it avoids single extreme values that may arise from misinterpretation of the image in one run. For categorical items, majority vote ensured that the final classification reflected the most frequent response across the three independent evaluations.

**Figure 2:**
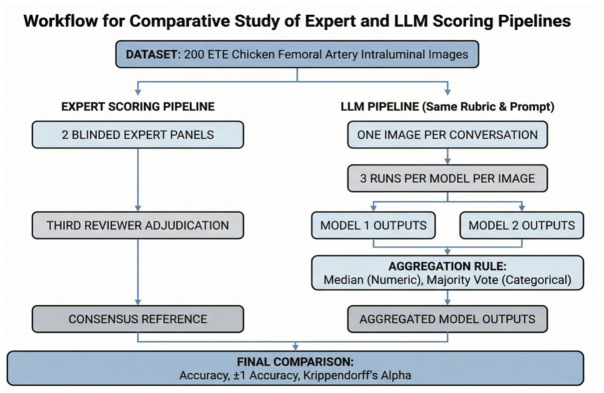
Study workflow (Legend: ETE = end-to-end; LLM = large language model).

The primary endpoint was exact-match agreement against the human expert analysis for each scored item. For the counting items, agreement within a tolerance of ±1 was also calculated. Inter-rater reliability was assessed using Krippendorff’s alpha, with the nominal metric used for categorical items and the ratio metric used for numeric count items. Agreements between each LLM and the human analysis were summarized for each endpoint, and three-rater agreement across LLM 1, LLM 2, and the human analysis was examined descriptively. Pairwise agreement between each LLM and the human analysis was summarized using simple percentage agreement, Light’s kappa, and Krippendorff’s alpha, as well as Bland–Altman analyses in the case of numeric count items [19,20]. Statistical analyses were performed using jamovi version 2.6.44 and R version 4.5.2 [21,22]. The detailed workflow is illustrated in Figure 2. Krippendorff’s alpha was selected as the primary reliability metric because it accommodates any number of raters, handles missing data, and applies to both nominal and ratio-level variables. Unlike Cohen’s kappa, which is limited to two raters and nominal categories, Krippendorff’s alpha permitted a unified analytical framework across all pairwise and three-rater comparisons. Bland–Altman plots were used for numeric items to visualize the magnitude and direction of counting differences between each LLM and the expert reference.

## 3. Results

Across all scored items, LLM 1 achieved a higher overall exact-match agreement than LLM 2 (0.659 vs. 0.539). For both models, performance was better on categorical than on numeric items (LLM 1: 0.713 vs. 0.610; LLM 2: 0.651 vs. 0.445).

Item-level performance is shown in Table 1. Among numeric items, Gaps showed the largest absolute advantage for LLM 1, with an exact-match agreement of 0.671 compared with 0.307 for LLM 2, followed by Knots (0.473 vs. 0.281) and Oblique stitches (0.446 vs. 0.317). Wide bites also favored LLM 1 (0.381 vs. 0.286), as did Partial-thickness bites (0.568 vs. 0.487). For Tears, both models achieved near-perfect agreement (0.995 and 0.990, respectively) on a dataset in which this finding was infrequent. Among categorical items, Wall catch yielded the highest agreement for both models (0.973 and 0.915), followed by Tension (0.969 and 0.920). Thread in lumen showed moderate agreement for LLM 1 (0.738) and lower agreement for LLM 2 (0.678). Line disruption was correctly classified in 74.3% of cases by LLM 1 and 69.3% by LLM 2. Overlap grade had the lowest categorical agreement for both models (0.470 and 0.407). Bite size showed a relatively small difference between models (0.598 vs. 0.563).

Krippendorff’s alpha showed the same overall pattern. LLM 1 had positive alpha values for most items, with the highest agreement for Wall catch (α = 0.432), Thread in lumen (α = 0.394), Tension (α = 0.385), Gaps (α = 0.256), Knots (α = 0.245), Line disruption (α = 0.234), Oblique stitches (α = 0.219), Overlap grade (α = 0.208), and Wide bites (α = 0.157). Agreement was near zero or slightly negative for Tears (α = 0.000), Partial-thickness bites (α = −0.013), and Bite size (α = −0.149). In contrast, LLM 2 had negative alpha values for all items, ranging from −0.248 for partial-thickness bites and −0.203 for wide bites to −0.00253 for tears.

When all three raters—LLM 1, LLM 2, and the expert reference—were analyzed together, agreement remained low across items (nominal α range, −0.0788 to 0.166; ratio α range, −0.0582 to 0.0462). For categorical items, the three-rater nominal α ranged from –0.079 for Bite size to 0.166 for Wall catch. For numeric count items, the three-rater ratio α ranged from –0.058 for Gaps to 0.046 for Wide bites.

For numeric items, allowing a tolerance of ±1 improved performance compared with strict exact matching, especially for Gaps, Knots, and Oblique stitches. Specifically, ±1 tolerance raised agreement from 0.671 to 0.971 for Gaps, from 0.473 to 0.846 for Knots, and from 0.446 to 0.944 for Oblique stitches in LLM 1. For LLM 2, the corresponding improvements were from 0.307 to 0.763 for Gaps, from 0.281 to 0.659 for Knots, and from 0.317 to 0.864 for Oblique stitches. Wide bites improved from 0.381 to 0.771 for LLM 1 and from 0.286 to 0.848 for LLM 2, while Partial-thickness bites rose from 0.568 to 0.802 and from 0.487 to 0.821, respectively. Tears reached perfect ±1 agreement for both models.

After aggregation across three runs, all final “Not assessable” classifications came from LLM 1, whereas LLM 2 produced only two isolated run-level “Not assessable” outputs, neither of which persisted after aggregation. Mean processing time was approximately 3 min for LLM 1 and 30 s for LLM 2. The higher frequency of “Not assessable” outputs from LLM 1 resulted in a reduced analyzable sample for certain items, most notably Wide bites (*n* = 105 for LLM 1 vs. 199 for LLM 2) and Partial-thickness bites (*n* = 162 vs. 199).

**Figure 3:**
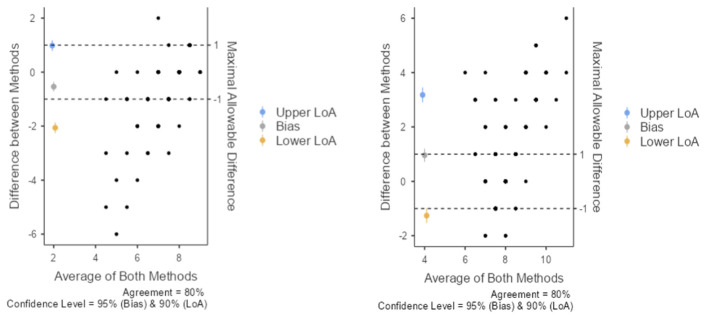
Bland–Altman analysis for knot counts: LLM 1 versus expert reference (**left**) and LLM 2 versus expert reference (**right**).

To further characterize disagreement, Bland–Altman analysis was performed for every item of each LLM and the human expert reference (Figure 3). For Knot counting for LLM 1, the mean difference was −0.538, with limits of agreement from −2.060 to 0.983 and a concordance correlation coefficient of 0.357. The negative mean difference indicates that LLM 1 tended to underestimate the number of knots relative to the expert reference, with most deviations falling within approximately one knot of the expert count. Bland–Altman analysis for knot counting by LLM 2 (Figure 3) revealed a contrasting pattern in which the mean difference was positive, indicating a tendency toward overestimation of knot counts relative to the expert reference. Both Bland–Altman plots also showed wider scatter at higher mean knot counts.

## 4. Discussion

Using the same prompt, rubric, and three-run aggregation strategy, ChatGPT 5.2 Thinking showed better agreement with expert scoring than Gemini 3.1 Pro. The clearest advantage was seen for numeric items requiring fine counting, such as gaps, knots, oblique stitches, and wide bites. By contrast, both models performed better on simpler categorical items such as tension and tears.

The Krippendorff’s alpha pattern adds important context to the values. For Tears, the near-zero alpha likely reflects the very low prevalence of this finding rather than true disagreement. In contrast, the consistently negative alpha values for LLM 2 suggest systematic disagreement with the expert reference, while the low alpha for Bite size and Overlap grade indicates persistent difficulty with fine visual classification.

The marked improvement in numeric-item agreement after allowing a ±1 tolerance suggests that many discrepancies were minor counting deviations rather than major miscounts. However, the absolute level of agreement remained insufficient for tasks requiring strict enumeration. In addition, the higher rate of “Not assessable” outputs from ChatGPT reduced the analyzable sample for some items, particularly Wide bites and Partial-thickness bites, and may have introduced a degree of selection toward clearer images.

Bland–Altman analysis showed a directional difference between the models: LLM 1 tended to underestimate knot counts, whereas LLM 2 tended to overestimate them relative to the expert reference. This suggests model-specific counting behavior rather than purely random variation. The wider scatter at higher mean knot counts also indicates that counting became more difficult as the number of stitches increased.

**Figure 4:**
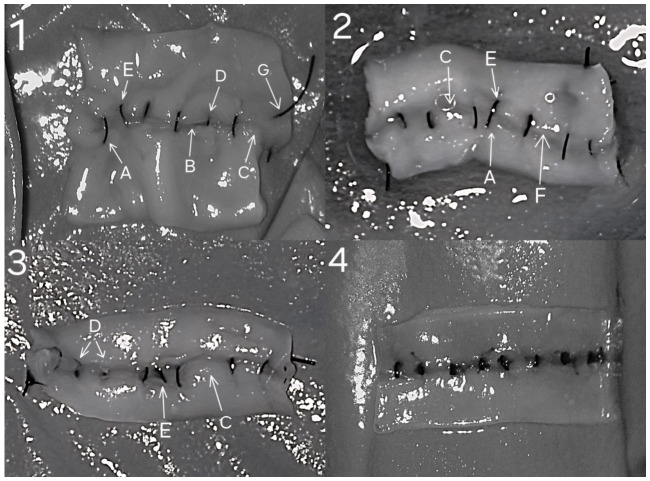
(**1**–**3**) Anastomoses with errors; (**4**) Error-free anastomosis. Legend: (A) Wide/large bite, (B) Tissue overlap, (C) Large distance between 2 knots, (D) Partial thickness bite, (E) Oblique stitch, (F) Disruption of anastomosis line, (G) Thread in lumen.

Because both models were evaluated with the same fixed prompt and rubric, the resulting differences are more likely to reflect the model performance within this workflow than prompt engineering variations. Errors seen in an anastomosis and an error-free anastomosis are illustrated in Figure 4.

These findings should also be viewed in the context of prior AI work in microsurgery. Most earlier studies focused on motion analysis, instrument tracking, hand landmark detection, or custom computer vision pipelines rather than direct end-product scoring from intraluminal images [13,23,24,25,26]. In contrast, the present study tested general-purpose vision-capable LLMs within a scoring framework. That makes the approach more accessible and easier to deploy, but it also highlights its current limits, particularly for exact counting and fine geometric discrimination. The advantage of using general-purpose LLMs is that they require no specialized training data, no custom model development, and no computational infrastructure beyond standard web access. This substantially lowers the barrier to entry for research groups and training centers that wish to incorporate AI-assisted assessment. However, the trade-off is that such models lack the domain-specific features that purpose-built systems can provide, which likely accounts for the disagreement observed in this study.

These results do not support replacing experienced microsurgeons in end-product assessment, particularly when precise evaluation is required, but they do point to a practical assistive role. Within a human-in-the-loop workflow, the LLM would perform initial scoring on all images, and the expert microsurgeon would review only those cases where the model’s output falls below a confidence threshold or where scores are different across repeated runs. Such selective verification could substantially reduce reviewer workload while preserving the accuracy of the final dataset, although its feasibility and efficiency remain to be tested prospectively. In this context, ChatGPT’s higher rate of “Not assessable” outputs may be viewed as a functional advantage because a conservative response pattern under uncertainty aligns with a broader principle in clinical assessment, where acknowledging ambiguity is preferable to producing a potentially inaccurate score. A model that explicitly flags ambiguous cases can direct expert attention toward the images that genuinely require human judgment, at the cost of a smaller analyzable dataset—a trade-off that must be weighed against the gain in reliability.

Future systems designed specifically for this task will likely need more than general visual reasoning. Efficient intraluminal microsurgical scoring would require stronger spatial recognition from 2D images, more reliable counting, and greater stability across repeated runs on identical inputs. The most effective future workflow would probably combine standardized model scoring with targeted expert verification, especially for low-confidence cases. Such a design could reduce reviewer workload without removing the need for expert analysis. From a practical standpoint, the processing time difference between the two models is also relevant to deployment decisions. ChatGPT’s approximately 3-min processing time per image, while substantially faster than manual expert review, may become a limiting factor when evaluating large batches of anastomoses in high-volume training settings. Gemini’s 30-s processing time offers a clear throughput advantage, but at the cost of lower agreement with expert consensus. An ideal deployment strategy might therefore consist of using the faster model for initial screening and reserving the more deliberate model for cases flagged as uncertain.

This study has several limitations. It evaluated a single imaging perspective and a rubric tailored to that setting. The findings may not generalize to other microsurgical tasks or to other video-based assessments without prior validation. In addition, repeated runs were used to reduce run-to-run variation, but a comparison of each run was not quantified. Finally, LLM performance may change over time as model versions and platform implementations evolve. Furthermore, the study was limited to a single type of anastomosis (end-to-end) performed on a single vessel model (chicken femoral artery), and the findings may not directly translate to other configurations such as end-to-side anastomoses or to clinical vessels with different tissue characteristics. The use of only two LLMs also limits the generalizability of the comparative findings, as other models with different architectures may yield different agreement profiles.

## 5. Conclusions

In this dataset of intraluminal images from end-to-end chicken femoral artery anastomoses, vision-capable LLMs showed only partial ability to approximate microsurgical end-product scoring. Performance was stronger for categorical findings and weaker for tasks that required exact counting or finer geometric discrimination. LLM-based scoring may be useful for expert review, but it cannot currently replace expert human analysis for precise microsurgical assessment. Future efforts should explore model-specific calibration, ensemble approaches combining multiple LLM outputs, and hybrid workflows in which AI-generated preliminary scores are selectively verified by expert reviewers, particularly for items involving fine spatial counting.

## Figures and Tables

**Table 1:** Performance analysis. LLM = large language model. LLM1 = ChatGPT 5.2 Thinking Extended (OpenAI). LLM2 = Gemini 3.1 Pro (Google). Num = numeric. Cat = categorical. n = number of cases included after excluding “Not assessable” values for the respective LLM. Agr. = exact-match agreement versus adjudicated expert consensus. α = Krippendorff’s alpha; nominal alpha was used for categorical items and ratio alpha for numeric count items, calculated on the same analyzable paired set used for the agreement comparison. ±1 Agr. = proportion of numeric predictions within ±1 of the expert consensus value. *p* = The two *p*-value columns are the Light’s Kappa *p*-values for each separate LLM–human pairwise analysis. “–” = not applicable.

Item	Type	n LLM1	Agr. LLM1	α LLM1	±1 Agr. LLM1	*p* LLM1 vs. Human	n LLM2	Agr. LLM2	α LLM2	±1 Agr. LLM2	*p* LLM2 vs. Human
Gaps	Num	173	0.671	0.256	0.971	0.002	199	0.307	−0.164	0.763	0.170
Knots	Num	182	0.473	0.245	0.846	<0.001	199	0.281	−0.087	0.659	0.811
Oblique stitches	Num	177	0.446	0.219	0.944	0.011	199	0.317	−0.062	0.864	0.306
Wide bites	Num	105	0.381	0.157	0.771	0.008	199	0.286	−0.203	0.848	0.694
Partial-thickness bites	Num	162	0.568	−0.013	0.802	0.253	199	0.487	−0.248	0.821	0.735
Tears	Num	194	0.995	0.000	1.000	1.000	199	0.990	−0.002	1.000	0.994
Line disruption	Cat	191	0.743	0.234	–	0.002	199	0.693	−0.119	–	0.678
Bite size	Cat	169	0.598	−0.149	–	0.577	199	0.563	−0.201	–	0.992
Thread in lumen	Cat	191	0.738	0.394	–	<0.001	199	0.678	−0.132	–	0.233
Wall catch	Cat	187	0.973	0.432	–	0.185	199	0.915	−0.042	–	0.939
Overlap grade	Cat	164	0.470	0.208	–	<0.001	199	0.407	−0.175	–	0.937
Tension	Cat	191	0.969	0.385	–	0.210	199	0.920	−0.0393	–	1.000

## Data Availability

The original contributions presented in this study are included in the article/supplementary material. Further inquiries can be directed to the corresponding author.

## Supplementary Materials

The following supporting information can be downloaded at: https://www.mdpi.com/article/10.3390/medsci14020235/s1, Supplementary File S1 (Item definitions) and Supplementary Table S2 (Whole Prompt).
